# Enhanced Expression of QTL *qLL9/DEP1* Facilitates the Improvement of Leaf Morphology and Grain Yield in Rice

**DOI:** 10.3390/ijms20040866

**Published:** 2019-02-17

**Authors:** Xue Fu, Jing Xu, Mengyu Zhou, Minmin Chen, Lan Shen, Ting Li, Yuchen Zhu, Jiajia Wang, Jiang Hu, Li Zhu, Zhenyu Gao, Guojun Dong, Longbiao Guo, Deyong Ren, Guang Chen, Jianrong Lin, Qian Qian, Guangheng Zhang

**Affiliations:** State Key Laboratory of Rice Biology, China National Rice Research Institute, Hangzhou 310006, China; fuxuezsy@163.com (X.F.); xujing87@126.com (J.X.); zhoumy@mail.sustc.edu.cn (M.Z.); jiashf@126.com (M.C.); baishushenlan@126.com (L.S.); 13258376009@163.com (T.L.); zyc1205926704@sina.com (Y.Z.); wgwangjiajia@163.com (J.W.); hujiang588@163.com (J.H.); zhuli05@caas.cn (L.Z.); zygao2000@hotmail.com (Z.G.); dongguojun@caas.cn (G.D.); guolongbiao@caas.cn (L.G.); rendeyongsd@163.com (D.R.); chenguang0066@126.com (G.C.); ljr1970@hotmail.com (J.L.)

**Keywords:** *Oryza sativa* L., leaf shape, yield trait, molecular breeding, hybrid rice

## Abstract

In molecular breeding of super rice, it is essential to isolate the best quantitative trait loci (QTLs) and genes of leaf shape and explore yield potential using large germplasm collections and genetic populations. In this study, a recombinant inbred line (RIL) population was used, which was derived from a cross between the following parental lines: hybrid rice Chunyou84, that is, *japonica* maintainer line Chunjiang16B (CJ16); and *indica* restorer line Chunhui 84 (C84) with remarkable leaf morphological differences. QTLs mapping of leaf shape traits was analyzed at the heading stage under different environmental conditions in Hainan (HN) and Hangzhou (HZ). A major QTL *qLL9* for leaf length was detected and its function was studied using a population derived from a single residual heterozygote (RH), which was identified in the original population. *qLL9* was delimitated to a 16.17 kb region flanked by molecular markers C-1640 and C-1642, which contained three open reading frames (ORFs). We found that the candidate gene for *qLL9* is allelic to *DEP1* using quantitative real-time polymerase chain reaction (qRT-PCR), sequence comparison, and the clustered regularly interspaced short palindromic repeat-associated Cas9 nuclease (CRISPR/Cas9) genome editing techniques. To identify the effect of *qLL9* on yield, leaf shape and grain traits were measured in near isogenic lines (NILs) NIL-*qLL9*^CJ16^ and NIL-*qLL9*^C84^, as well as a chromosome segment substitution line (CSSL) CSSL-*qLL9*^KASA^ with a Kasalath introgressed segment covering *qLL9* in the Wuyunjing (WYJ) 7 backgrounds. Our results showed that the flag leaf lengths of NIL-*qLL9*^C84^ and CSSL-*qLL9*^KASA^ were significantly different from those of NIL-*qLL9*^CJ16^ and WYJ 7, respectively. Compared with NIL-*qLL9*^CJ16^, the spike length, grain size, and thousand-grain weight of NIL-*qLL9*^C84^ were significantly higher, resulting in a significant increase in yield of 15.08%. Exploring and pyramiding beneficial genes resembling *qLL9*^C84^ for super rice breeding could increase both the source (e.g., leaf length and leaf area) and the sink (e.g., yield traits). This study provides a foundation for future investigation of the molecular mechanisms underlying the source–sink balance and high-yield potential of rice, benefiting high-yield molecular design breeding for global food security.

## 1. Introduction

Rice leaf morphogenesis and its spatial extension posture are important components of ideal plant architecture, which play a significant role in the photosynthetic efficiency and grain yield [1,2]. During the grain-filling stage, the top three leaves, particularly the flag leaf, are the main carbohydrate sources that were transported to panicles and grains for yield formation. The establishment of better source-to-sink biomass allocation would greatly contribute to the improvement of rice yield potential [3,4]. Therefore, using molecular genetic techniques to modulate the top three leaf morphology and improve the photosynthesis rate so as to balance the relationship with the grain sink will effectively achieve a high yield in rice.

The polarity development of leaves along the adaxial–abaxial, the medial–lateral, and the apical–basal axis determines the construction of the three-dimensional spatial morphology of the leaf. About 40 genes related to leaf morphogenesis have been cloned, and studies have mainly focused on leaf width, length, and rolling. Rice leaf width is mainly related to the number of veins and the distance between veins, which are regulated by the following aspects: microRNA shear-related genes, such as *OsDCL1* [5] and *GIF1* [6]; cell division-related genes, such as *SRL2* [7], *SLL1*/*RL9* [8,9], *OsCCC1* [10], and *OsWOX4* [11]; *NAL2* /*NAL3* [12], *SLL1* [8], *OsCD1* [13], and genes related to coding transcription factors and cellulases; *NAL1*/*LSCHL4* [14,15], *NAL7* [16], *TDD1* [17], *OsCOW1* [18], *OsARF19* [19], and genes associated with auxin synthesis and metabolism; and genes including *NAL9* encoding an ATP-dependent Clp protease proteolytic subunit [20]. In a series of cloned rolling genes in rice, the type I genes, such as *ADL1* [21], *OsAGO7* [22], *OsAGO1a* [23], *SLL1* [8], and *RL9* [9], regulate the unbalanced development of different tissues in the adaxial/abaxial side, which affects the curl degree of blades. The type II genes are associated with the development of bulliform cells in the adaxial side, and changes in the size or amount affect the curl degree of the blade. Adaxially and abaxially rolled leaves appear as favored by various genes, such as *ACL1* [24], *LC2*/*OsVIL3* [25,26], *OsCOW1*/*NAL7* [16,18], *OsCD1*/*NRL1*/*ND1*/*sle1*/*DNL1* [13,14,27,28,29], *OsHox32* [30], *OsMYB103L* [31], *OsZHD1* [32], *REL1* [33], *REL2* [34], *RL14* [35], *ROC5* [36], *CLD1* [37], *SRL1* [38] *SFL1* [39], *SLL2* [40], *YABBY1* [41], and *LRRK1* [42]. The type III genes, such as *SLL1* [8], *RL9* [9], *SRL2* [7], *AVB* [43], and *OsSND2* [44], control the development of sclerenchyma in the abaxial side and affect the curl degree of blades. The genes of type IV, such as *CFL1* [45], include those with an abnormal cuticle development, leading to leaf curl. The genes of type V, such as *CVD1*, regulate commissural veins (CVs), and the lack of CV in *cvd1* mutant is the main cause of leaf curl [46]. 

The length, width, and area are the three traits determining the shape and size of a leaf, which are quantitatively inherited. Using a DH (doubled haploid) population, Li et al. detected two major QTLs for the flag leaf length, which were located on chromosome 4 and chromosome 8, respectively [47]. Yan et al. studied the genotype–environment interaction of eight plant morphological traits using a DH population and mapped seven QTLs on chromosomes 1, 2, 3, 4, 6, 9, and 10 related to the length of the flag leaf [48]. Farooq et al. identified three leaf length QTLs on chromosomes 1, 2, and 4 using IR64 derived introgression lines [49]. Although the important role of leaf traits in plant ideotype in rice has attracted great attention, the cloning of QTLs for leaf length is rarely documented.

The coordinated balance of source and sink is an essential component to ensure a high yield in rice. Notably, genetic populations used for leaf traits were generally among those used for yield traits, where QTLs for leaf traits were frequently located in regions in which QTLs for yield traits were detected [50,51,52,53,54,55,56,57,58]. The major leaf width QTL *qFLW4/LSCHL4/SPIKE* allelic to *NAL1* is related to both leaf morphology and yield traits [15,58,59]. Similarly, the pleiotropic effect on leaf morphology regulation was also found in the cloning of rice grain-shaped QTL. Large-grain alleles in the *GS2* locus simultaneously increase leaf length [60,61]. However, in rice high-yield breeding, studies have yet to be conducted on the influence of the leaf shape alleles/QTLs from different donors in the interaction of molecular level between the regulation of leaf morphogenesis and yield formation. As reported, up to 50% of the variation in panicle weight depended on the variation in leaf size [50]. The co-location of QTLs/genes for source–sink traits in rice could increase the source while expanding the sink, provided that the QTLs have the same effect direction for both traits, which will be invaluable genetic resources for breeding high-yield varieties [62].

In this study, QTL mapping for leaf length and width of the top three leaves in rice was performed at two different environments using a recombinant inbred lines (RILs) set derived from the cross between maintainer line and restorer line of the *indica–japonica* super hybrid rice Chunyou 84, followed by validation and fine mapping. The target major QTL *qLL9* controlling leaf length was delimitated into a 16.17 kb interval between markers C-1640 and C-1642 on chromosome 9, using a residual heterozygote identified from the RIL population, which were segregated at *qLL9* with high homogenous backgrounds. Then, gene cloning, functional analysis, and breeding potential assessments of *qLL9* were conducted.

## 2. Results

### 2.1. Analysis of Leaf Morphology in the RIL Population and Their Parental Cultivars 

**Figure 1:**
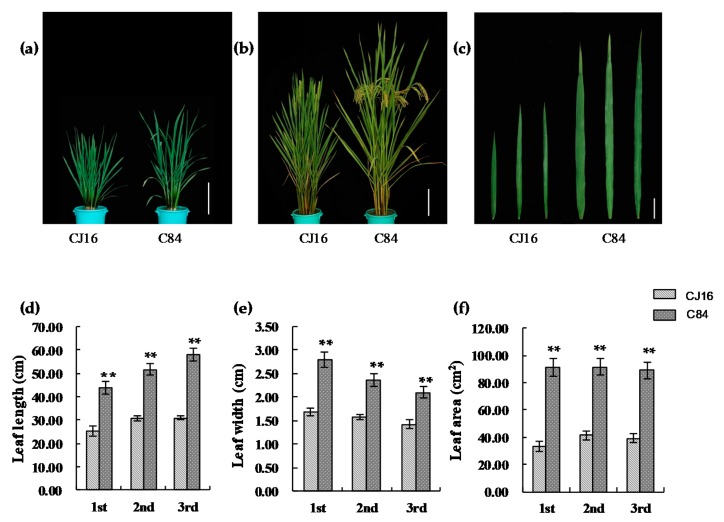
The leaf shape of parents of recombinant inbred lines (RILs). (**a**) Plant morphology at tillering stage; bar = 18 cm. (**b**) Plant morphology at heading stage; bar = 18 cm. (**c**) The top three leaves’ shape of CJ16 and C84. From left to right are the first leaf, the second leaf, and the third leaf, respectively; bar = 5 cm. (**d**) Comparison of the top three leaves’ length between CJ16 and C84. (**e**) Comparison of the top three leaves’ width between CJ16 and C84. (**f**) Comparison of the top three leaves’ area between CJ16 and C84. Data are represented as mean ± SD (*n* = 11). Asterisks represent significant difference determined by Student’s *t*-test at *p*-value <0.01 (**), *p*-value <0.05 (*).

**Figure 2:**
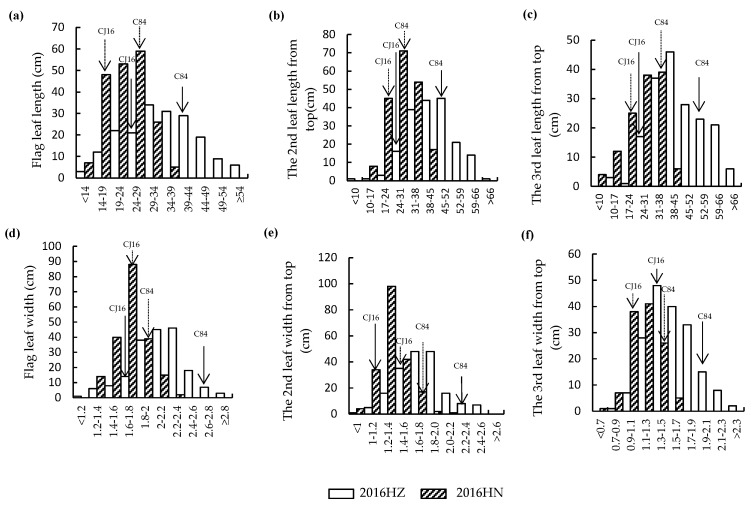
Frequency distributions of leaf traits in CJ16/C84 RILs. (**a**) Flag leaf length; (**b**) the second leaf length from top; (**c**) the third leaf length from top; (**d**) flag leaf width; (**e**) the second leaf width from top; (**f**) the third leaf width from top. HZ: Hangzhou; HN: Hainan.

The morphology of the top three leaves of RIL parents Chunjiang 16B (CJ16) and Chunhui 84 (C84) was investigated and significant differences were found for leaf length, width, and area (Figure 1a–c). Compared with CJ16, the top three leaves length of C84 were 42.2%, 40.2%, and 46.6% longer, respectively (Figure 1d). Similarly, the leaf width of C84 was significantly wider than that of CJ16 (Figure 1e). Thus, a much larger leaf area was found in C84, that is, 2.7 times the flag leaf and 2.2 times both the second and third leaves (Figure 1f). In RIL population, the leaf traits of the top three leaves were all continuously distributed with large variations and transgressive segregation, showing a typical pattern of quantitative variation at both Hangzhou and Hainan experimental sites (Figure 2), which were suitable for QTL mapping. Furthermore, we observed that the leaf traits of RILs in Hangzhou generally tended to higher values, while those in Hainan tended to lower ones.

### 2.2. Correlation Analysis and QTL Mapping 

The correlation analysis for leaf traits in the RIL population showed a low correlation between Hangzhou and Hainan for the length of the second and the third leaf. However, the flag leaf length and width, and the second and third leaf width, were significantly positively correlated between the two environments. This could be the result of the *indica–japonica* subspecies differentiation of the parents and the distinct temperature and light conditions of Hangzhou and Hainan (Table 1).

**Figure 3:**
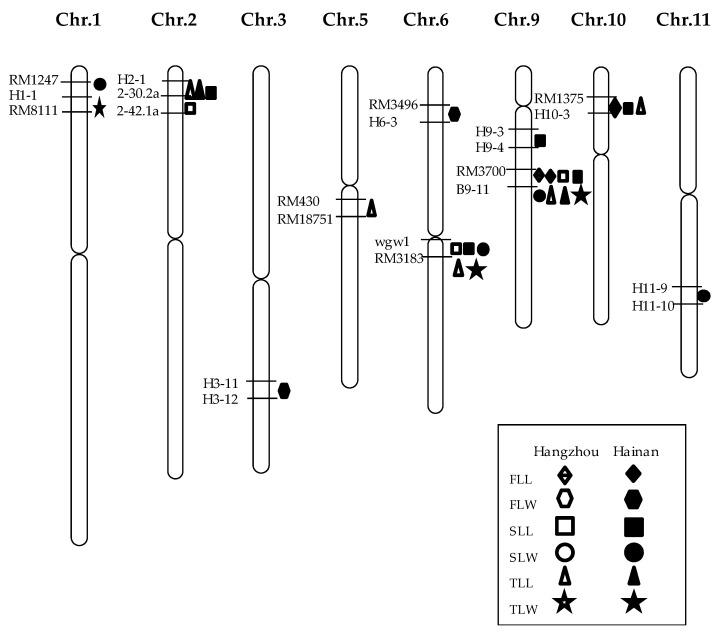
Locations of quantitative trait loci (QTLs) for leaf traits in the genetic map. FLL: flag leaf length; FLW: flag leaf width; SLL: the second leaf length; SLW: the second leaf width; TLL: the third leaf length; TLW: the third leaf width.

QTL mapping was performed for the leaf length and width of the top three leaves (Table 2 and Figure 3). The results showed that a total of 27 QTLs were detected in the two environments, which were distributed on chromosomes 1, 2, 3, 5, 6, 9, 10, and 11. In Hangzhou, nine QTLs were detected, including one QTL for the flag leaf length, three QTLs for the second leaf length, and five QTLs for the third leaf length, which explained phenotypic variance in the range of 7.76%–32.41 %. In Hainan, 18 QTLs were detected, including two QTLs for the flag length, two QTLs for the flag leaf width, five QTLs for the second leaf length, four QTLs for the second leaf width, two QTLs for the third leaf length, and three QTLs for the third leaf width, which explained phenotypic variance in the range of 1.85%–30.63%. Among them, three leaf length QTLs, namely, the flag leaf length QTL *qFLL9*, the second leaf length QTL *qSLL9*, and the third leaf length QTL *qTLL9*, were simultaneously mapped in RM3700-B9-11 interval on chromosome 9 across the two environments, with the enhancing alleles all from C84, explaining phenotypic variance ranging from 19.19% to 32.41%, which agreed with the significantly positive correlation of flag leaf length between Hangzhou and Hainan (Table 1). Meanwhile, even though no significant correlation was found for the leaf length of the second and third leaf, consistent QTLs across both locations were also detected, that is, *qSLL6* and *qTLL2*, which could be because of their larger genetic effects and/or less sensitivity to environmental variation. In addition, we noted QTLs for the width of the second and third leaf were also detected in the interval between RM3700 and B9-11, but with the increasing alleles coming from CJ16. These results prompt us to mainly focus on the major region flanked by RM3700 and B9-11 on chromosomes 9, which showed stable effects on leaf morphological development, and named *qLL9*.

### 2.3. Fine Mapping and Leaf Shape Characterization of qLL9 

**Figure 4:**
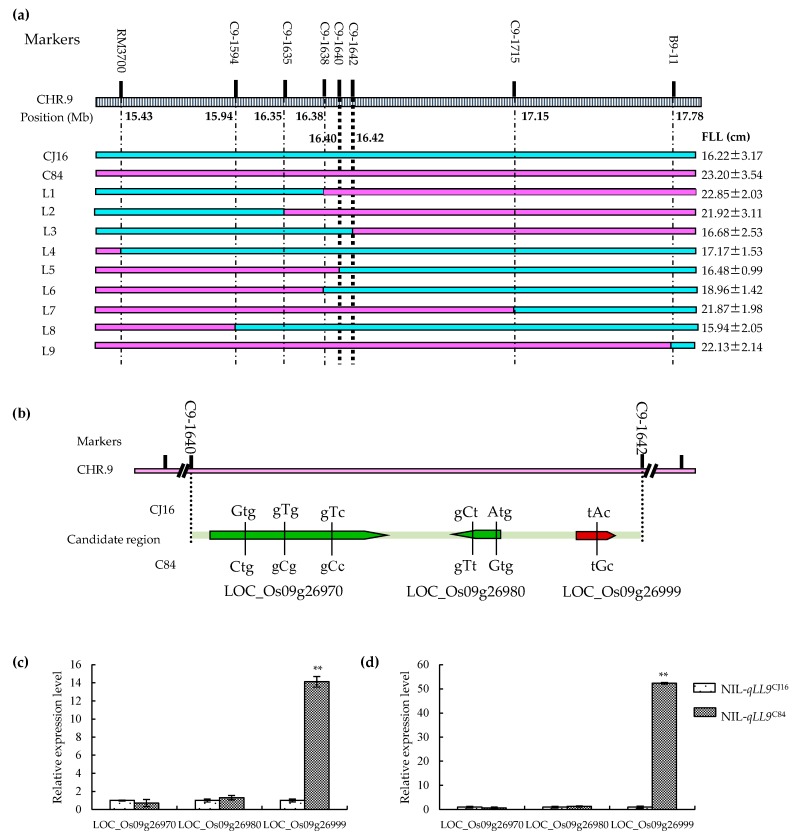
Map-based cloning of *qLL9* and expression analysis of candidate genes. (**a**) High-resolution mapping of *qLL9*. Numbers on the map indicate the physical distance on chromosome 9. Nine recombinant plants were used to refine the candidate region to 16.17 kb region by substitution mapping, in which green and pink rectangles indicate the homozygous CJ16 genotype and homozygous C84 genotype, respectively. Flag leaf length (FLL) values were obtained from the corresponding selfed progenies and represented as mean ± SD (*n* = 12). (**b**) Predicted open reading frames and sequence difference of the *qLL9* between CJ16 and C84 are shown. Relative expression of three candidate genes *LOC_09g26970*, *LOC_09g26980*, and *LOC_09g26999* of near isogenic line (NIL)-*qLL9*^CJ16^ and NIL-*qLL9*^C84^ at tillering stage (**c**) and heading stage (**d**) by quantitative real-time polymerase chain reaction (qRT-PCR). Data are mean ± SD (*n* = 3). Asterisks represent significant difference determined by Student’s *t*-test at *p*-value < 0.01 (**).

According to the flanking markers RM3700 and B9-11 on chromosome 9, one residual heterozygote with a heterozygous segment covering the interval was identified from the RILs, from which a large population was derived. Nine representative near isogenic lines (NILs) with introgressions covering different portions of the target region were identified using DNA markers in the target interval for QTL fine mapping (Table 3; Figure 4a). Then, combined with their flag leaf length, *qLL9* was delimitated into a 16.17 kb region between markers C9-1640 and C9-1642 (Figure 4a). 

**Figure 5:**
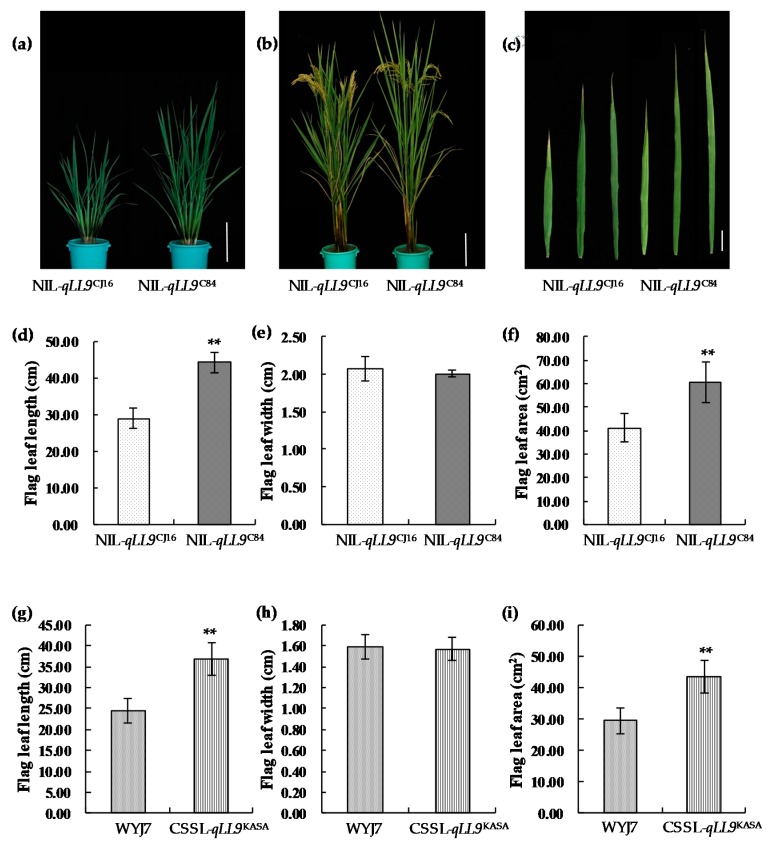
Phenotypes of near-isogenic lines and chromosome segment substitution lines. (**a**) Plant morphology at tillering stage; bar = 18 cm. (**b**) Plant morphology at heading stage; bar = 18 cm. (**c**) The top three leaf shape of NIL-*qLL9*^CJ16^ and NIL-*qLL9*^C84^. From left to right are the first leaf, the second leaf, and the third leaf, respectively; bar = 5 cm. Comparison between the NIL-*qLL9*^CJ16^ and NIL-*qLL9*^C84^ for the flag leaf length (**d**), the flag leaf width (**e**), and the flag leaf area (**f**). Comparison between Wuyunjing (WYJ) 7 and chromosome segment substitution line (CSSL)-*qLL9*^KASA^ for the flag leaf length (**g**), the flag leaf width (**h**), and the flag leaf area (**i**). Data are mean ± SD (*n* = 15). Asterisks represent significant difference determined by Student’s *t-*test at *p*-value < 0.01 (**).

To further clarify the effect of *qLL9* on leaf morphology, we compared the leaf traits in a near-isogenic line set of NIL-*qLL9*^CJ16^ and NIL-*qLL9*^C84^, and a chromosome segment substitution line (CSSL)-*qLL9*^KASA^ and its recurrent parent Wuyunjing (WYJ) 7. In both groups, significant differences for the flag leaf were only found for the length, but not the width (Figure 5), while both the leaf length and width variation were significant for the second and third leaf from the top (Figure S1), which were highly consistent with the findings in our QTL primary mapping (Table 2). Obviously, compared with NIL-*qLL9*^CJ16^, the longer flag leaf length of NIL-*qLL9*^C84^ resulted in a larger flag leaf area (Figure 5d–f). However, the opposite allelic effects on leaf length and width of the second and third leaf from the top even made the leaf area variation not significant (Supplementary Figure 1). Similarly, after introgression of the Kasalath allele at *qLL9* in WYJ 7, CSSL-*qLL9*^KASA^ resulted in a 1.5 times longer flag leaf from 29.4–43.5 cm^2^ (Figure 5g–i).

**Figure 6:**
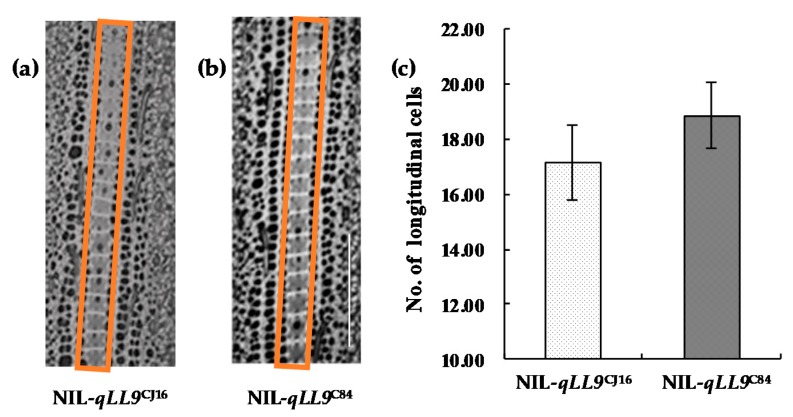
Histological cell morphology of NIL-*qLL9*^CJ16^ and NIL-*qLL9*^C84^. (**a**,**b**) Comparison of cytological morphological characteristics in the orange boxes between the two genotypes of the NIL set; bar = 200 μm. (**c**) Number of longitudinal cells in NIL-*qLL9*^CJ16^ and NIL-*qLL9*^C84^. Data are mean ± SD (*n* = 15) and no significant difference was found.

On the other hand, it is noted that the flag leaf epidermal cells showed no significant difference in either the size or number in unit area between NIL-*qLL9*^CJ16^ and NIL-*qLL9*^C84^, which suggested the variation of flag leaf length could be mainly attributed to the increase of the total cell number (Figure 6). In summary, *qLL9* influenced the leaf length and area variation by regulating the development of leaf cells.

### 2.4. Determination of the Candidate Gene

According to the database of Rice Genome Annotation Project (http://rice.plantbiology.msu.edu), three open reading frames (ORFs) were predicted in the target region defined by C9-1640 and C9-1642 (Figure 4b and Table 4), namely, LOC_Os09g26970 encoding cytochrome P450 protein, LOC_Os09g26980 encoding retrotransposon protein, and LOC_Os09g26999 encoding G protein γ subunit (a cloned gene *DEP1*) [63]. By sequencing and qRT-PCR for the three candidates, six non-synonymous single nucleotide polymorphisms (SNPs) were found across the three candidate genes (Figure 4b and Table 5). No significant difference was detected in the LOC_Os09g26970 and LOC_Os09g26980 expression between NIL-*qLL9*^CJ16^ and NIL-*qLL9*^C84^ at both the tillering and heading stages, but the expression of LOC_Os09g26999 in NIL-*qLL9*^C84^ was significantly increased by 14.1 times and 52.3 times, respectively, compared with NIL-*qLL9*^CJ16^ at the two stages (Figure 4c,d). Therefore, LOC_Os09g26999 could be the best potential candidate for *qLL9.* In addition, we found that *qLL9* was allelic to the known gene *DEP1* [63]. We inferred that the difference of amino acid of *qLL9* would have an influence on the gene expression and would thus affect the leaf morphological development.

**Figure 7:**
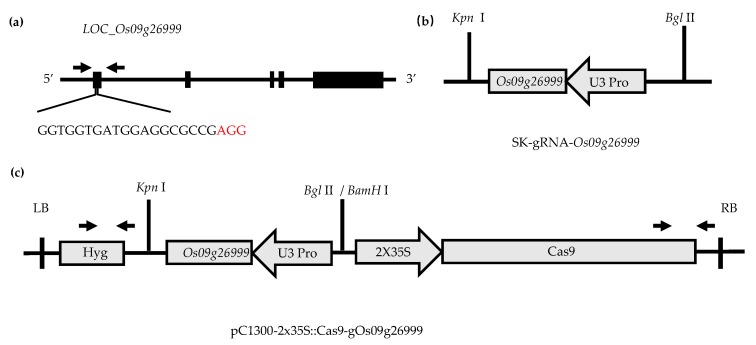
CRISPR/Cas9 vector construction. (**a**) Primer sequence on *LOC_Os09g26999*. (**b**) The intermediate vector SK-gRNA contains the U3 promotor and sgRNA scaffold. (**c**) Binary vector pC1300-Cas9 contains the 2 × 35S promotor and a Cas9 protein. SK-gRNA-*Os09g26999* are digested with *Kpn* I and *Bgl* II, respectively, and cloned into pC1300-Cas9 (digested with *Kpn* I and *BamH* I) by a one-step ligation.

**Figure 8:**
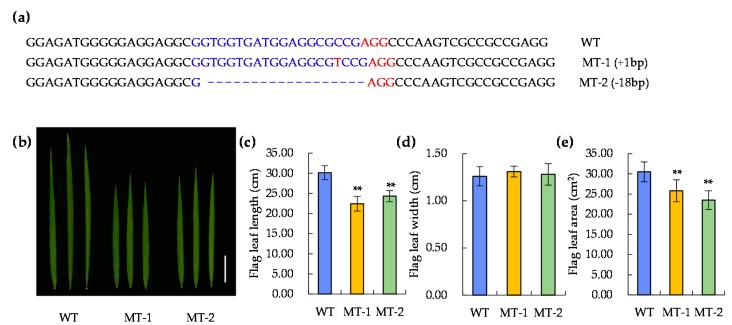
Sequence of target loci in CRISPR/Cas9 transgenic plants and phenotypic comparison between the knockout and wild-type (WT) plants. (**a**) Sequence variation of target loci in two transgenic plants. (**b**) Flag leaf variation; bar = 5 cm. Difference in (**c**) flag leaf length (cm), (**d**) flag leaf width (cm), and (**e**) flag leaf area. WT, Nipponbare; MT-1, Mutant 1; MT-2, Mutant 2. Data are represented as mean ± SD (*n* = 15). Asterisks represent significant difference determined by Student’s *t*-test at *p*-value <0.01 (**).

It has been reported that *DEP1* is mainly related to panicle development [63]. To further verify its correlation with leaf development, the CRISPR/Cas9 gene knockout technique was adopted in the genetic background of Nipponbare (Figure 7). Two knockout plants of LOC_Os09g26999, namely mutant 1 (MT-1) and mutant 2 (MT-2), were screened in T0 generation (Figure 8; Figure S2). After continuous selfing and marker assay, homozygous positive transgenic lines in T2 generation were obtained and used for investigating the flag leaf morphology. The results showed no significant difference in the flag leaf width between the knockout and wild-type plants. However, the flag leaf length and area were significantly decreased in both knockout lines. (Figure 8b–e). The results showed that *qLL9* was mainly responsible for the leaf length development, and the loss-function of *qLL9* could lead to a reduction in leaf length, and thereby leaf area.

**Figure 9:**
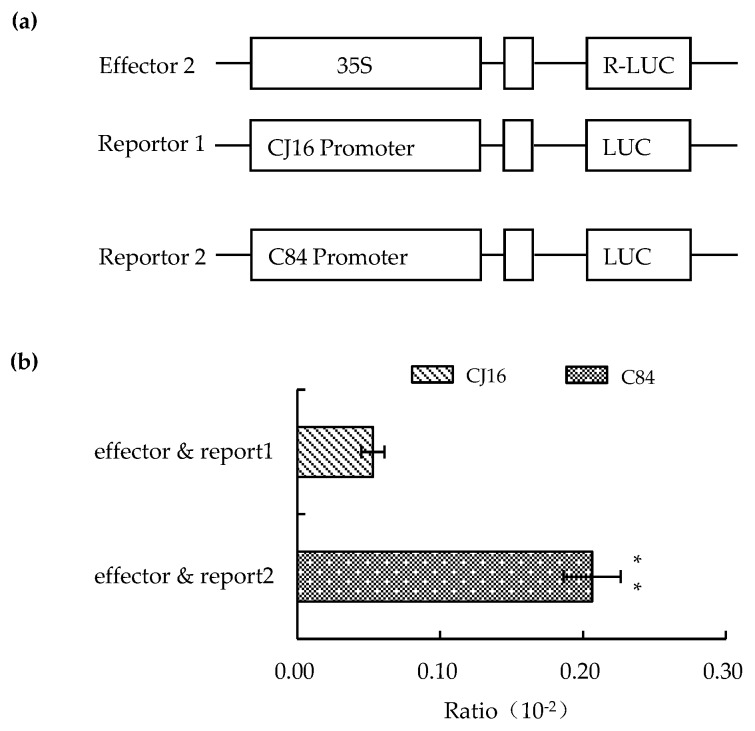
Comparison of the promoter activity of LOC_Os09g26999 between CJ16 and C84. (**a**) Diagram of carrier construction. (**b**) Ratio of promoter activity. Data are represented as mean ± SD (*n* = 3). Asterisks represent significant difference determined by Student’s *t*-test at *p*-value <0.01 (**).

To further explore the causal factor for differential expression of *LOC_Os09g26999* observed in the NIL set, we compared the promoter sequence of 2.0 kb upstream of the ATG between them, and found nine variations (Table 6). Then, the activity difference of the two types of promoters was compared by dual luciferase reporter assay. The result showed that the LUC-C84 promoter had 3.9 times higher expression of reporter genes than the LUC-CJ16 promoter (Figure 9). That is to say, the promoter activity of *qLL9* also played a significant role in the gene expression and leaf-trait variation.

### 2.5. qLL9 Affecting the Yield Traits

**Figure 10:**
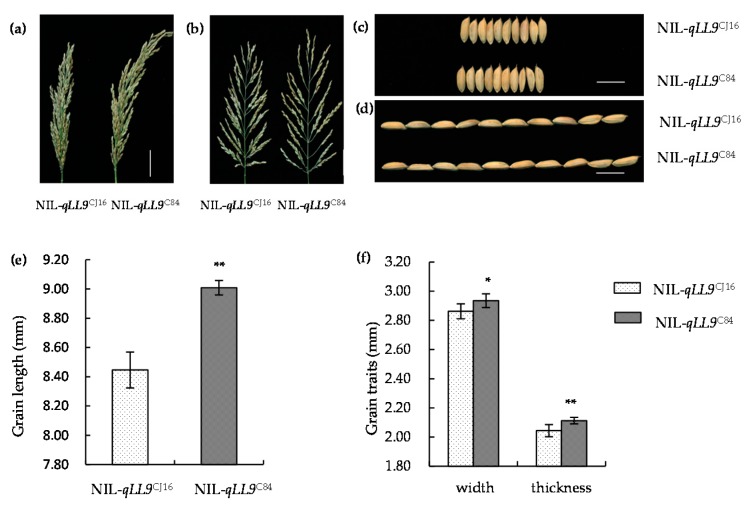
Panicle and grain morphology of NIL-*qLL9*^CJ16^ and NIL-*qLL9*^C84^. (**a**,**b**) Panicle morphology at heading stage; bars = 4 cm. (**c**,**d**) Grain morphology at repining stage; bar = 1 cm. (**e**) Grain length. (**f**) Grain width and thickness. Data are represented as mean ± SD (*n* = 10). Asterisks represent significant difference determined by Student’s *t*-test at *p*-value <0.01 (**), *p*-value <0.05 (*).

To verify whether *qLL9* affected the yield formation, we firstly investigated its effects on grain shape using NIL-*qLL9*^CJ16^ and NIL-*qLL9*^C84^ plants (Figure 10). We found that the grain length, width, and thickness of NIL-*qLL9*^C84^ are all slightly larger than those of NIL-*qLL9*^CJ16^ by 6.66%, 2.55%, and 3.37%, respectively.

Then, we compared the other yield component traits between the NIL-*qLL9*^CJ16^ and NIL-*qLL9*^C84^ plants. There was no significant difference between them in the number of productive panicles per plant, the number of primary branches per panicle, the number of second branches per panicle, and the number of grains per panicle (Table 7). However, the thousand-grain weight of NIL-*qLL9*^C84^ was significantly higher than that of NIL-*qLL9*^CJ16^ (22.99 g and 20.79 g, respectively), while the seed setting rate of NIL-*qLL9*^C84^ was lower (69.71% and 75.80%, respectively). Even so, the yield per plant of NIL-*qLL9*^C84^ was 16.59% higher than that of NIL-*qLL9*^CJ16^. That is, the increase in the yield per plant was mainly attributed to the increase in thousand-grain weight. Further, yield measurement in plots also showed that the NIL-*qLL9*^C84^ could yield more grains than NIL-*qLL9*^CJ16^, increasing by 15.08%. As for the actual yield per hectare, NIL-*qLL9*^C84^ could increase by 991.67 kg compared with NIL-*qLL9*^CJ16^. These results showed that the C84 allele at *qLL9* significantly increases the grain size, thousand-grain weight, and grain yield in the field production.

## 3. Discussion

In this study, the main effect QTL *qLL9* related to the leaf length was positioned by RIL population. *LOC_Os09g26999* was identified as the target gene through sequence comparison, expression analysis, and CRISPR-Cas9 gene editing technology. It is allelic to the known spike-shaped gene *DEP1/EP/qPE9-1* [63,64,65]. The plants of NILs and CSSLs carrying different alleles of *qLL9* showed great differences in leaf, spike, and yield. All results indicated that QTL *qLL9* has a pleiotropic function in rice. In addition to regulating spike development, *qLL9* is also plays a key role in the development of leaf morphology, grain shape, yield, and other traits.

Previous studies have demonstrated that QTL *qLL9* candidate gene *LOC_Os09g26999* encodes a cysteine-rich region [66]. Huang et al found that the replacement of a 637 bp stretch from the fifth exon of *LOC_Os09g26999* with a 12 bp sequence results in erect panicle architecture because of the early termination of translation [63]. Due to the differences of *LOC_Os09g26999* relative expression and promoter activity in CJ16 and C84, the promoter sequences and regulatory elements were compared by website (https://sogo.dna.affrc.go.jp). We found that nine nucleotide differences were observed among the parental lines, eight of which were located in cis-acting elements possibly by mediating enhancer activity depending on upstream region G/C mutation at 1255 bp upstream of ATG, which is consistent with site II transcriptional core sequence regulatory elements (TGGGCC^CJ16^ to TGGCCC^C84^). It plays an important role in the specific expression of *proliferating cell nuclear antigen* (*PCNA*) gene in rice meristem [67,68]. We speculate that G/C mutation may be through mediation of enhancer activity dependent on far upstream regions. Comparison of CDS showed that a single base substitution event is observed from A to G at 3,182 bp downstream of ATG in *LOC_Os09g26999* between CJ16 and C84, leading to the substitution of amino acids from tyrosine to cysteine. This event might lead to changes in the structure of the γ subunit, affecting the signal transduction of G protein. Further work is needed to determine whether the difference expression of *LOC_Os09g26999* is due to changes in protein structure or differences in promoter activity. 

**Figure 11:**
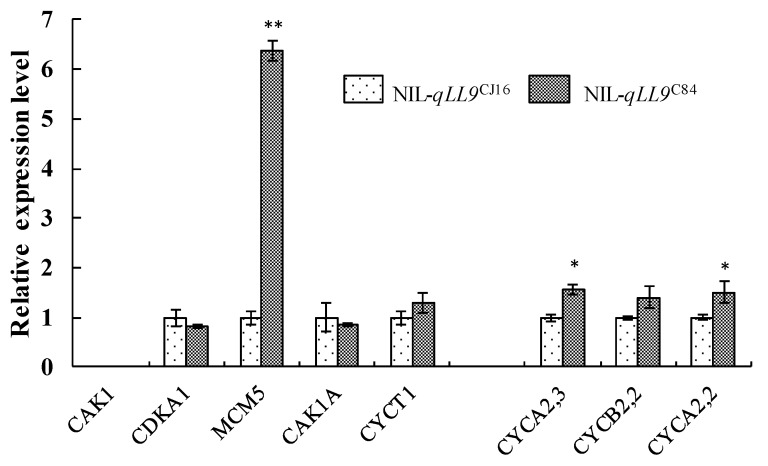
Comparison of eight cell cycle related gene expression between NIL-*qLL9*^CJ16^ and NIL-*qLL9*^C84^ at the heading stage by qRT-PCR, and the data are represented as mean ± SD (*n* = 3); asterisks represent significant difference determined by Student’s t-test at p-value <0.01(**), p-value <0.05 (*).

The leaf morphological development in rice is regulated by the size and number of leaf epidermal cells. In *rot4-1D* mutant, the reduction in the number of leaf longitudinal cells induces a short leaf [69]. Tsuge et al revealed that the *Arabidopsis thaliana rotundifolia3* leaf mutant has the same number of cells as the wild type, but with reduced cell elongation in the leaf-length direction [70]. Rice flag leaf width QTL *qFLW7*, homologous to Arabidopsis *LNG1*, regulates the longitudinal growth of cells and its overexpression results in elongated leaves [71]. In this study, no significant difference was observed in the cell size per unit area of the leaf epidermis between NIL-*qLL9*^C84-^ and NIL-*qLL9*^CJ16^, whereas the total number of cells increased leading to leaf longer. The expression levels of eight cell cycle-related genes in NILs were analyzed at the heading stage (Figure 11 and Supplementary Table S1), which showed that the expression of *MCM5* plays an important role in the initiation and extension of DNA replication in the G1 phase, which was significantly up-regulated in NIL-*qLL9*^C84^ compared to NIL-*qLL9*^CJ16^ [72]. Plant Class A cyclin (Cyclin) *CYCA2;3* and *CYCA2;2* [73,74] could identify and interact with different cyclin-dependent kinases, which were also remarkably up-regulated in NIL-*qLL9*^C84^. Therefore, *qLL9* from *indica* rice C84 could improve the DNA replication efficiency of leaf tissue cells, accelerate cell division, and promote leaf elongation, which also corresponded to the fact that the morphology of the leaf epidermal cells of NIL-*qLL9*^C84^ was unchanged while the total number of the cells increased. These findings indicated that *qLL9* may affect plant morphological development by participating in the regulation of cell division cycle. Notably, the regulatory mechanisms of different genes on leaf morphological development are different, thus their interactions in leaf morphogenesis should be studied in details in the future.

## 4. Materials and Methods

### 4.1. RIL Population and Field Trial

The RIL population consisting of 188 lines was derived from the cross of the *japonica* rice Chunjiang 16B (CJ16) as the female parent and the *indica* rice Chunhui84 (C84) as the male parent, which are the maintainer and restorer lines of the commercial intersubspecific hybrid rice Chunyou84. The rice population was tested at experimental fields of the China Rice Research Institute located in Hangzhou, Zhejiang, and Lingshui, Hainan, during May–October 2016 and November 2016–April 2017, respectively. Twenty-five-day-old seedlings were transplanted at a hill spacing of 20 cm × 20 cm with three replications. In each replication, one line was grown in three-row plots with six plants per row. The block was managed in accordance with conventional field management, and diseases, insects, and weeds were controlled [75]. The leaf morphology and yield traits were investigated at the heading stage and mature stage. In each trial, data of the three replications were averaged for each line and used for data analysis.

### 4.2. Statistical and Genetic Analysis 

The linkage map of the RILs consisted of sixty-nine simple sequence repeats (SSR) and eighty-nine sequence tagged site (STS) DNA markers. QTL analysis was conducted using QTL Network 2.1. Critical *F* values for genome-wise type I error were calculated with 1000 permutation tests and used for claiming a significant event. A significant level of *p* < 0.005 was set for candidate interval selection, putative QTL detection, and QTL effect estimation. The proportion of phenotypic variance (*R^2^*) explained by a single main QTL for a given trait in a given population was calculated by Markov Chain Monte Carlo algorithm. In the genome scan, a testing window of 10 centimorgan (cM), filtration window of 10 cM, and walk speed of 1 cM were chosen. The naming of QTLs was based on the method of McCouch et al. [75].

### 4.3. Map-Based Cloning and Candidate-Gene Promoter Activity for qLL9

Using flanking markers RM3700 and B9-11 of *qLL9* obtained from QTL mapping, residual heterozygotes (RHs) were screened from the RIL population, which were segregated at *qLL9* with high homogenous genetic background. Six pairs of molecular markers were developed in this target interval (Table 3). By substitution mapping, we investigated the flag leaf length and the new developed marker genotypes of 2307 RH-derived introgression lines, and then the *qLL9* was fine mapped. 

### 4.4. RNA Extraction and qRT-PCR

Total RNA of NIL-*qLL9*^CJ16^ and NIL-*qLL9*^C84^ was extracted from the penultimate leaves at the tillering stage and the flag leaf at heading stage to analyze the expression difference of candidate genes (Table 3 and Table 4). We used the AxyPrepTM Multisource Total RNA Miniprep Kit (Axygen) to extract total RNA, which was then retro-transcribed using PrimeScriptTM RT Reagent Kit with gDNA Eraser (Takara, Dalian, China). Quality and concentration of the RNA extracted were checked with electrophoresis on 1% agarose gel and measured using the Nanodrop ND-2000 spectrophotometer (NanoDrop Technologies, Wilmington, CA, USA). Concentration of the RNA samples used for cDNA synthesis was normalized by dilution with RNase-free ultra-pure water. qRT-PCR assays of 20 μL reaction volumes, which contained 0.5 μL of synthesized cDNA, 0.4 μM of gene-specific primers, and 10 μL of SYBR^®^ Premix Ex Taq^TM^ (Takara), were conducted using ABI 7500 Real-time PCR System (Applied Biosystems, Foster, CA, USA). Following the manufacturer’s instruction, the qRT-PCR conditions were set up as follows: denaturing at 95 °C for 30 s, then 40 cycles of 95 °C for 5 s, 55 °C for 30 s, and 72 °C for 30 s. To standardize the quantification of gene expression, we used the rice *Ubiquitin* (*UBQ*) gene (Os03g0234200, http://rapdb.dna.affrc.go.jp/) as an internal control.

According to the sequence shown in http://rice.plantbiology.msu.edu/index.shtml, the primer POs26999-F/R (Table 3) was used to amplify LOC_Os09g26999 promoter of CJ16 and C84, which were constructed into *pGreenII0800-LUC* using homologous recombination [76]. Positive clones were screened by colony PCR and sequencing and were named as *proCJ16-LUC* and *proC84-LUC*. The plasmids and the internal reference (R-LUC) were transformed into the protoplasts of rice variety 93-11 by 40% PEG-3350 (Sigma, St. Louis, MO, USA) solution-mediated transformation [77]. The dual-luciferase reporter gene detection kit (Promega Company, Madison, WI, USA) was used for detection and analysis of the promoter activity.

### 4.5. CRISPR/Cas9 Transgene Analysis

The target sequence of the potential candidate LOC_Os09g26999 refers to a previous study [78]. Synthetic primers Os26999-g++ and Os26999-g−−were used to make the target site adapter (Table 3). This adapter was connected to an SK-gRNA carrier, and positive intermediate carrier SK-gRNA-Os26999 was screened out. pC1300-2×35S::Cas9-gOs26999 final expression carrier was constructed by means of enzyme digestion-joining method. *Agrobacterium tumefaciens* EHA105 was transformed through positive cloning. *Agrobacterium tumefaciens*-mediated gene transfer experiments were carried out with the background of Nipponbare [79]. The result was analyzed via the sequential decoding method (http://dsdecode.scgene.com/) to identify transgenic positive plants. At heading stage, 15 wild-type and 15 mutant plants were selected to measure the length, width, and area of the flag leaf.

### 4.6. Construction of Near Isogenic Lines and Chromosome Segment Substitution Lines and Trait Measurement

Using flanking markers C-1640 and C-1642 (Table 3), a set of near isogenic lines (NILs) for *qLL9* was identified from RH-derived segregating populations with a higher homogenous background, named NIL-*qLL9*^CJ16^ and NIL-*qLL9^C8^*^4^. At the same time, one chromosome segment substitution line (CSSL) for *qLL9* was obtained with Kasalath (KASA) as the donor parent and Wuyunjing 7 (WYJ 7) as the recurrent parent through one cross followed by six continuous backcrosses and one self-crossing, named CSSL-*qLL9^KASA^*. 

The leaf length, width, and area of the NIL and CSSL set were investigated at the heading stage, while the panicle length, the number of primary and secondary branches, the number of grains per panicle, grain length, grain width, grain thickness, thousand-grain weight, and setting percentage were scored from 10 randomly selected main panicles at maturity. Then, Student’s *t*-test was adopted to analyze the phenotypic difference between the two genotypic groups in each set.

The grain yield was measured using the NIL set NIL-*qLL9*^CJ16^ and NIL-*qLL9^C8^*^4^, of which each was planted in three 48 m^2^ plots, at a hill spacing of 20 cm × 20 cm. At maturity, three points in each plot were randomly harvested, with 30 hills per point, to determine the grain yield, which is then converted into the grain yield per hectare.

### 4.7. Morphological Observation on Leaf Epidermal Cytology

The commercially available transparent nail polish without color is selected, which is conducive to the transparency of microscopic materials. The flag leaves of tested rice plants at the heading stage were sampled and painted with the nail polish evenly at the same part for 10 min air-dry. When an open mouth exists at the end of the coating layer of the nail polish, the dried coating is torn with a transparent tape and placed on the fragment. Then, the cover slip was covered. The filter paper was covered by the blunt end of the dissecting needle, and the fragment was gently pressed to make a temporary filling piece. Under the electron microscopy, the cell size and number of 15 leaves of a single plant were analyzed [80].

## 5. Conclusions

Our work identified the genetic contribution of *qLL9* to both flag leaf morphologic development and yield formation. Improving the photosynthetic efficiency and coordinating the source–sink interaction through leaf morphogenesis are the premise to increase the rice yield and to establish the ideal plant type. Identification and utilization of the QTLs with beneficially pleiotropism for both leaf shape and grain yield would greatly contribute to molecular breeding of superior rice. For example, the QTL *qLSCHL4*/*NAL1* is correlated with leaf shape and involved in regulating the development of chlorophyll content, grain number and grain weight [15]. QTL *qFLW7^9311^* related to flag leaf width, could improve leaf shape and grain traits, remarkably increase rice yield in the field [71]. Similarly, *GS2* could increase both grain weight/size and leaf length and thus grain yield [60,61]. According to the main objectives of rice molecular design breeding, future studies should systematically analyze the source-sink relationship and the genetic network of plant type establishment, directional polymerize the *qLL9, NAL1, qFLW7, GS2,* and other beneficial genes from *indica* and *japonica* subspecies of rice to improve the yield potential and establish the ideal plant architecture in molecular breeding of superior rice.

## Figures and Tables

**Table 1:** Correlation analysis on leaf traits in recombinant inbred lines (RILs) derived from the cross of CJ16 and C84.

	HZ						HN				
	FLL	FLW	SLL	SLW	TLL	TLW	FLL	FLW	SLL	SLW	TLL
HZ-FLL											
HZ-FLW	−0.078										
HZ-SLL	0.833 **	−0.013									
HZ-SLW	−0.178 *	0.627 **	−0.072								
HZ-TLL	0.525 **	0.000	0.746 **	−0.023							
HZ-TLW	−0.203 **	0.597 **	−0.073	0.699 **	0.086						
HN-FLL	0.341 **	−0.317 **	0.271 **	−0.202 *	0.336 **	−0.252 **					
HN-FLW	−0.250 **	0.389 **	−0.298 **	0.382 **	−0.287 **	0.246 **	0.076				
HN-SLL	0.225 **	−0.268 **	0.135	−0.137	0.195 *	−0.226 **	0.873 **	0.229 **			
HN-SLW	−0.418 **	0.390 **	−0.510 **	0.441 **	−0.395 **	0.361 **	−0.021	0.754 **	0.160		
HN-TLL	0.000	−0.182 *	-0.098	−0.087	0.007	−0.132	0.642 **	0.254 **	0.813 **	0.313 **	
HN-TLW	−0.405 **	0.246 **	−0.477 **	0.337 **	−0.355 **	0.300 **	0.088	0.624 **	0.206 *	0.760 **	0.358 **

FLL: flag leaf length; FLW: flag leaf width; SLL: the second leaf length; SLW: the second leaf width; TLL: the third leaf length; TLW: the third leaf width. Data are represented as mean ± SD (*n* = 3). Asterisks represent significant difference determined by Student’s *t*-test at *p*-value < 0.01 (**), *p*-value < 0.05 (*).

**Table 2:** Locations of quantitative trait loci (QTLs) for leaf traits in the RIL population.

Trait	QTL	Interval		Peak Position		Additive Effect		Explained Phenotypic Variance (%)	
		Marker 1	Marker 2	HZ	HN	HZ	HN	HZ	HN
**FLL**	*qFLL9*	RM3700	B9-11	61.1	56.87	−7.1707	−6.8682	32.41	30.63
	*qFLL10*	RM1375	H10-3		20.09		3.3926		5.09
**FLW**	*qFLW3*	H3-11	H3-12		152.3		0.0648		6.6
	*qFLW6*	RM3496	H6-3		27.14		−0.0839		16.07
**SLL**	*qSLL2-1*	2-30.2-a	2-42.1-a	36.2		−2.8643		8.98	
	*qSLL2-2*	H2-1	2-30.2-a		23.9		−1.3914		5.29
	*qSLL6*	wgw1	RM3183	59.4	60.1	3.5234	−4.2842	14.97	17.29
	*qSLL9*	RM3700	B9-11	60.1	57.6	−6.4659	−6.5431	29.62	20.85
	*qSLL9-2*	H9-3	H9-4		12.18		−0.7557		8.8
	*qSLL10*	RM1375	H10-3		27.11		3.9058		5.91
**SLW**	*qSLW1*	RM1247	H1-1		24.2		−0.0759		10.69
	*qSLW6*	wgw1	RM3183		59.35		−0.0727		10.31
	*qSLW9*	RM3700	B9-11		59.2		0.0839		18.39
	*qSLW11*	H11-9	H11-10		71.4		−0.0329		1.85
**TLL**	*qTLL2*	H2-1	2-30.2-a	13.0	19.44	−3.6555	−0.9935	13.10	8.99
	*qTLL5*	RM430	RM18751	70.3		−2.5759		8.43	
	*qTLL6*	wgw1	RM3183	35.2		−4.8389		15.65	
	*qTLL9*	RM3700	B9-11	59.1	61.2	−6.0826	−4.8326	21.87	19.19
	*qTLL10*	RM5689	RM1375	28.6		2.3523		7.76	
**TLW**	*qTLW1*	H1-1	RM8111		22.41		−0.0669		10.79
	*qTLW6*	wgw1	RM3183		59.35		−0.0684		13.84
	*qTLW9*	RM3700	B9-11		61.31		0.0834		16.28

**Table 3:** Primers for QTL fine mapping, quantitative real-time polymerase chain reaction (qRT-PCR), vector construction, and gene editing.

Primer	Forward (5′-3′)	Reverse (5′-3′)	Experiment
RM3700	AAATGCCCCATGCACAAC	TTGTCAGATTGTCACCAGGG	Fine mapping
C9-1594	CCTGTACACTGTAGGCCTGT	GGTGTCAAAGTACATAGGCCC	
C9-1635	GGTGGAAAGGAAGGAGAGCT	CTAGCCCTGCCTCGTTGTAA	
C9-1638	GTGTGTGTGTGTGTGTGTGT	TCATAGTACATGCCCTCCGT	
C9-1640	ATAAGTCCATATTGCCCACCTC	AAGCTTCTGGATCGTTAACAGG	
C9-1642	GTACCCTCCTCCGATGACAC	TTGTGGAGGACGAGAAGGTG	
C9-1715	GGTGGCGAGAAGAATTTGCA	TTTCGCCTCTCACTGACCTT	
B9-11	TCTTACGAATAGGCCCTTGG	AGAGCCCACAACACTTGTGC	
Actin	ATCCATCTTGGCATCTCTCAGC	CACAATGGATGGGCCAGACT	qRT-PCR
LOC_Os09g26960	CTGAGCCTCGCCAATCTG	CGAAGATCTCCTCCATGCTC	
LOC_Os09g26970	CAAACATCTGGGCTTGGTCT	TCTAAGCAACCTGCCCAATC	
LOC_Os09g26980	ATTGATGTGAAAGGGCAAGACT	CACCTTAAGCCCAAGGTTGTAG	
LOC_Os09g26999	GTAGCTGCAAGCCAAGCTG	TTGAAGCAGCTGGAGCAAC	
POs26999	GGCCAGTGCCAAGCTTAAGGGAAGTTGGCCGCCTGCC	AGGGTCTTGCAGATCTCTCCACACGCAGCACGCCAAC	Vector construction
Os09g26999-g++/g--	GGCAGGTGGTGATGGAGGCGCCG	AAACCGGCGCCTCCATCACCACC	
Os09g26999-JC	CGGCGATTTATACCCACCAC	CGCTCACCTTGAGGAACGT	Detection of target mutations
Hyg-F1	GCTGTTATGCGGCCATTGTC	GACGTCTGTCGAGAAGTTTC	
Cas9-F2/pC1300-R2	ACCAGACACGAGACGACTAA	ATCGGTGCGGGCCTCTTC	
T3	ATCGGTGCGGGCCTCTTC		

**Table 4:** Annotated genes included in the 16.17kb region for *qLL9*.

Gene ID	Annotation from the Rice Genome Annotation Project
LOC_Os09g26970	Retrotransposon protein, Putative, Unclassified, Expressed
LOC_Os09g26980	Cytochrome P450, Putative, Expressed
LOC_Os09g26999	Gγ subunit; Dense and Erect Panicle1; DENSE PANICLE 1

**Table 5:** The position of SNP and amino acid variation for the three candidate genes.

Locus Name	Position on chr.9	Position on gene	CJ16	C84	CJ16	C84
LOC_Os09g26970	16393266	397	G	C	Val	Leu
LOC_Os09g26970	16393717	848	T	C	Val	Ala
LOC_Os09g26970	16395923	3054	T	C	Val	Ala
LOC_Os09g26980	16403850	4146	G	A	Ala	Val
LOC_Os09g26980	16404094	3902	T	C	Met	Cys
LOC_Os09g26999	16414735	3182	A	G	Tyr	Cys

**Table 6:** The position of nine SNPs in the promoter of LOC_Os09g26999.

Position	CJ16	C84
−1341	A	C
−1255	G	C
−951	C	G
−906	T	G
−629	G	T
−503	A	C
−493	G	C
−486	T	G
−31	TG	T

**Table 7:** Yield traits of NIL-*qLL9*^CJ16^ and NIL-*qLL9*^C84^.

Trait	NIL-*qLL9*^CJ16^	NIL-*qLL9*^C84^
Panicle length (cm)	20.44 ± 0.84	26.7 ± 0.83 **
Panicles per plant	9.25 ± 1.42	9.42 ± 1.26
Number of primary branches	22.11 ± 2.18	21.4 ± 2.42
Number of secondry branches	80.00 ± 15.51	77.60 ± 7.84
Grains per panicle	404.11 ± 71.70	355.17 ± 34.09
1000-grain weight (g)	20.79 ± 0.74	22.99 ± 0.45 **
Seed setting rate (%)	75.80 ± 0.02	69.71 ± 0.02 **
Yield per plant (g)	27.93 ± 4.41	32.56 ± 8.19
Actual yield per plot (kg/48 m^2^)	31.52 ± 3.20	36.28 ± 3.27 *
Actual yield change (%)	–	15.08

Data are represented as mean ± SD. Asterisks represent significant difference determined by Student’s *t*-test at *p*-value <0.01(**), *p*-value <0.05 (*).

## Supplementary Materials

Supplementary materials can be found at http://www.mdpi.com/1422-0067/20/4/866/s1.
