# Supplementary material for: Enhanced Expression of QTL qLL9/DEP1 Facilitates the Improvement of Leaf Morphology and Grain Yield in Rice
Source: Int J Mol Sci. 2019 Feb 17;20(4):866. doi: 10.3390/ijms20040866 (PMC6412340; doi:10.3390/ijms20040866)
Supplement: Supplementary file 1 [file ijms-20-00866-s001.pdf]

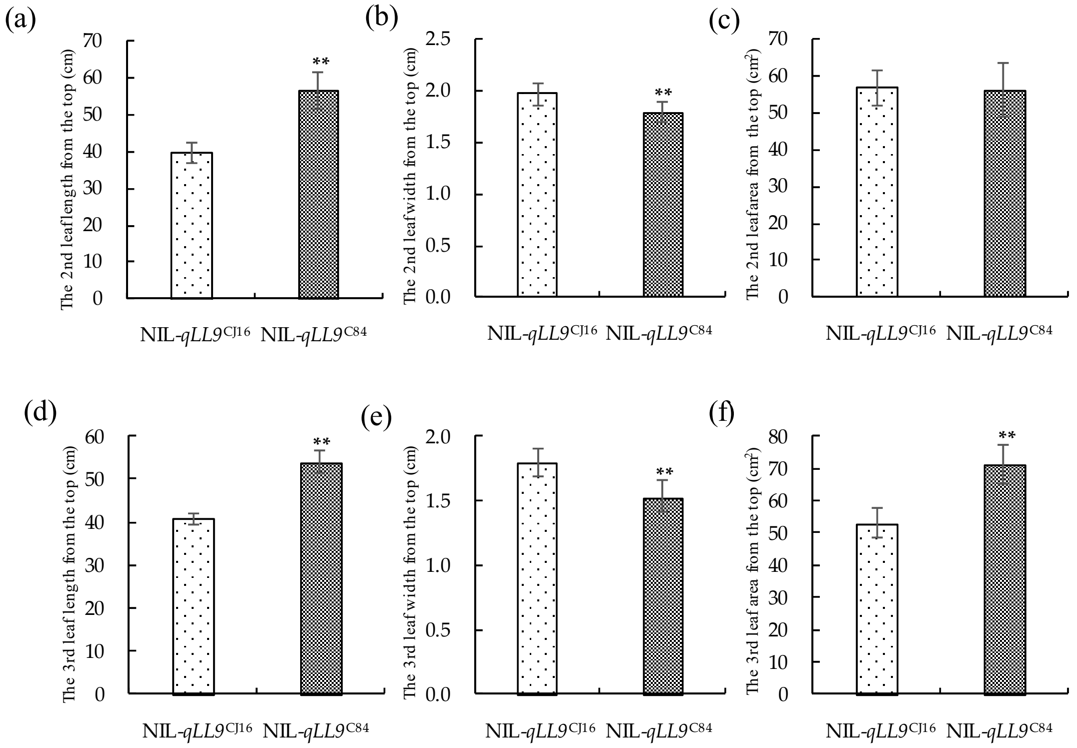

Supplementary Figure S1 Comparison between the NIL-*qLL9*^{CJ16} and NIL-*qLL9*^{C84} for the 2nd leaf length (d), the 2nd leaf width(e), the 2nd leaf area (f), the 3rd leaf length (d), the 3rd leaf width(e), and the 3rd leaf area (f). Data are represented as mean ± SD (n=15). Asterisks represent significant difference determined by Student's *t*-test at p-value<0.01(**), p-value<0.05 (*).

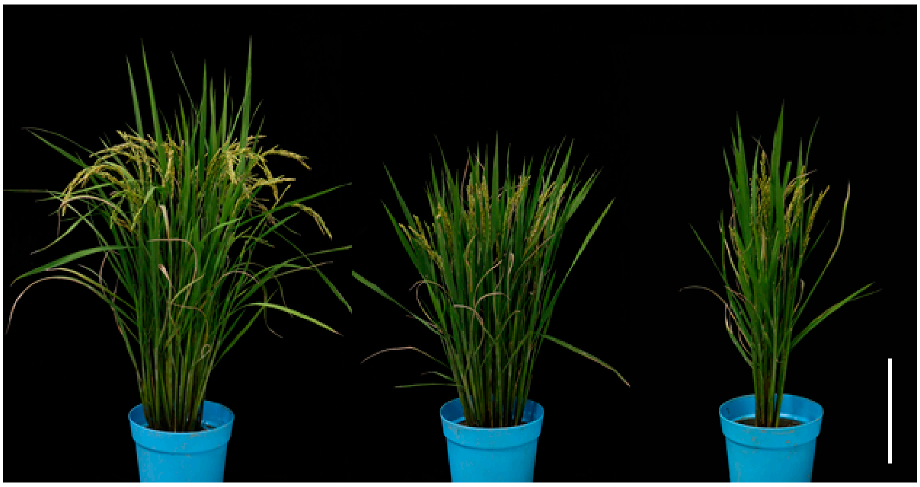

WT MT-1 MT-2

Supplementary Figure S2. Phenotypes of the CRISPR/Cas9 transgenic and wild-type plants. WT, Nipponbare; MT-1, Mutant 1; MT-2, Mutant 2. Bar=18 cm.

Supplementary Table S1. Primers for qRT-PCR of eight cell cycle related genes

Primer	Forward (5′-3′)	Reverse (5′-3′)
Actin	ATCCATCTTGGCATCTCTCAGC	CACAATGGATGGGCCAGACT
CAK1	GACGGTCAGATTAGACGCAAGA	TCCAAAGGATGTCCACA
CDKA1	GGTTTGGACCTTCTCTCTAAAATGC	AGAGCCTGTCTAGCTGTGATCCTT
CAK1A	GACCGACAAGGGTTTCAGCAT	CCAGCATGTTCAGGAAGATACAAT
MCM5	AAGGAGAACTGCCTGTCCATGA	AGTGGCCTTAGCTTTCACCCTC
CYCT1	GCATTTGTTGCAGCTCAAG	TCACCACTTCGCTGACTTATTG
CYCA2.2	AGGTTGTCAAGATGGAGAGCGA	CGCTTTTTGTCTTCCTGGCA
CYCA2.3	GTTTCGGTTGACGAGACGATGT	CGCTGCAAGGAACCTAGAACTG
CYCB2.2	CTCAAGGCTGCACAATCTGACA	GCATTGACGGCTGGAATTTG

.
